# The profile of persons with traumatic spinal cord injury and the associated factors of length of stay in a rehabilitation centre, in South Africa

**DOI:** 10.1038/s41393-026-01189-y

**Published:** 2026-03-03

**Authors:** Tammy-Lee Williams, Lena Nilsson Wikmar, Conran Joseph

**Affiliations:** 1 Department of Physiotherapy, Faculty of Community and Health Sciences, University of the Western Cape, Cape Town, South Africa; 2 Department of Neurobiology, Faculty of Care Sciences and Society, Karolinska Institute, Stockholm, Sweden; 3 Division of Physiotherapy, Faculty of Health and Rehabilitation Services, Stellenbosch University, Stellenbosch, South Africa

**Keywords:** Neurology, Spinal cord diseases

## Abstract

**Study design:**

Single-site retrospective cohort study based on medical records review from 2016–2020. Specialised rehabilitative care is required to integrate traumatic spinal cord injury (TSCI) survivors into the community. The World Health Organisation’s (WHO) Rehabilitation 2030 initiative calls to strengthen rehabilitation services, and therefore, information regarding the injury profiles and length of rehabilitation stay (LORS) may assist in planning rehabilitation.

**Objectives:**

The study aimed to examine the profile of individuals with TSCI, who were admitted to a rehabilitation centre, and to describe the factors associated with the LORS.

**Setting:**

Western Cape Rehabilitation centre, South Africa.

**Methods:**

The International Spinal Cord Injury Core Data Setversion 2.0 was used to extract data. One-way ANOVA and t-tests compared group means. A multiple linear regression analysis was done to account for possible intercorrelations between predictors.

**Results:**

The population consisted predominantly of males (89.8%) in the 31-–40-year-old category. Assault was the leading cause of injury (70.4%). The thoracic spine was most affected (52.4%), with complete injuries (AIS A, according to the International Standards for Neurological Classification of Spinal Cord Injury (INCSCI)) most common (42.8%). Cervical injuries were associated with significantly longer rehabilitation stays compared to thoracic or lumbar injuries.

**Conclusion:**

The high prevalence of assault-related etiology underscores the need for preventative strategies to reduce the incidence of TSCI among young males. The characteristics of this unique population and the factors associated with LORS have important implications for healthcare planning, particularly in optimising staff and bed resources, to enhance access to specialised care.

## Introduction

TSCI has a reported global incidence of 26.48 (95% CI, 24.15–28.93) per million [1]. In low- and middle-income countries (LMICs), the incidence of TSCI is as high as 22.55 per million, per year [2]. LMICs are defined as those countries with a below-average standard of living, income, economic and industrial development. As of 2024, 152 countries met the definition of a LMICs, including countries in central and South America, Africa, most of the Asian countries and many island states [3]. In Botswana, the annual incidence of TSCI was estimated at 13 per million [4]. In Cape Town, from 2013–2014, the crude incidence rate was estimated at 75.6 per million (95% CI: 64.3–88.8) amongst a government-funded healthcare system [5], and 20 per million in the private healthcare system [6].

With this high incidence of TSCI, and with its healthcare restraints such as understaffing, long patient waiting times and reduced resources, access to specialised care may be limited in South Africa [5]. Specialised acute and rehabilitative approach to the management of spinal cord injury (SCI) is required to improve functional outcomes and integrate survivors back into the community [7]; such care is available in developed countries such as Sweden, where the delivery of gold standard care in managing SCI survivors is standard [8]. In South Africa, the only specialised acute care unit in the Western Cape has a 2% mortality rate and an average length of stay (LOS) of 28 days for SCI survivors [9]. Previous reporting identified that LOS could be influenced by the occurrence of secondary health conditions, such as pressure ulcers and pulmonary complications, in both acute care and rehabilitation [10].

Improving universal health coverage involves healthcare planning, such as LOS (in acute and rehabilitation centres), financing strategies and health system strengthening with the aim of improving health efficiency [7]. LOS is often used as a measure of hospital management efficiency, with longer LOS indicating poorer use of resources and shorter LOS indicating efficient use of resources [8]. However, this is not entirely true as studies have shown that various factors, such as the development of complications, type of injury and severity of injury, affect LOS and not simply hospital management efficiency [10–12].

The current profile of persons with TSCI in Cape Town, South Africa, consists predominantly of young males with assault as the prevalent etiology [5]. Studies exploring epidemiological characteristics are important to provide insight into the causes of, and to identify who are at a greater risk for, TSCI. These studies aid in developing targeted preventative strategies. To date, epidemiological studies in Cape Town, South Africa are eight years and older [5, 9]. Although these studies are still pertinent for preventative measures, our study aims to add to this body of knowledge.

In South Africa, access to specialised rehabilitation services for individuals with TSCI is constrained by limited rehabilitation capacity, inequitable distribution of services across geographic regions, and insufficient integration of rehabilitation at primary care levels, resulting in many survivors not receiving the full continuum of care needed to optimise functional outcomes and community reintegration [13]. Given these limited resources, specifically in relation to rehabilitation, there is a need to examine factors related to LORS, which could aid in more equitable provision of rehabilitation for more individuals with TSCI. The findings of this study may assist in planning rehabilitation services and improve access to care, in line with the call for action to strengthen rehabilitation services worldwide through the ‘Rehabilitation 2030’initiative [14].

## Methods

### Study setting, design and procedure

A retrospective medical records review was conducted for the period of April 2016 – March 2020. The review took place at the Western Cape Rehabilitation centre (WCRC) located in Cape Town. WCRC is an in-patient and out-patient governmental centre providing specialised care to persons with disabilities [15]. The inclusion criteria consisted of all adults diagnosed with a TSCI and admitted during April 2016 – March 2020. G-Power [16] was used to determine the minimum sample to obtain a medium effect size (Cohen’s f2 = 0.15) with power = 0.80 and alpha level = 0.05 for multiple regression model with 11 predictors, which was *n* = 123. G-Power also indicated that the actual achieved power with a sample size of n = 625 was 1.00.

The International Spinal Cord Injury Core Data Set, version 2.0 [17] was used to extract data from the medical records. The data included demographic information, date of injury, cause of injury (sports, assault, transport etc.), details of injury (spinal region affected, vertebral injury, spinal surgery), date of admission and discharge from rehabilitation (length of rehabilitation stay), and the ISNCSCI, specifically the AIS classification, to describe the neurological status of individuals.

### Statistical analysis

Participant characteristics (age, gender, year of injury), injury characteristics (cause of injury, spinal region affected, AIS classification) and the LORS were analysed descriptively. One way-ANOVA was used to determine the differences in means for more than two known groups, while a t-test was used when there were only two known groups. To account for the possible intercorrelations between predictors (LORS, gender, age, vertebral injury, surgery, spinal region affected, ISNCSCI), all associated factors were entered into a multiple linear regression analysis.

Prior to interpreting the results obtained through the statistical analysis, we verified that the assumptions of the statistical tests were met. Concerning the ANOVA and t-test, we used Levene’s F-test to verify the assumption of homogeneity of variance. In the case of the t-test, equality of variance between those who had surgery and those who did not was denoted by the Levene’s F-test, (F = 0.005, *p* = 0.492) including those who had a vertebral injury and those who did not (F = 3049, *p* = 0.06). In ANOVA, Levene’s F-test demonstrated that the assumption of homogeneity of variance had been violated for the spinal region affected groups (F = 15.33, *p* < 0.001) and thus the Games-Howell post-hoc test for unequal variances was used to compare the groups. In the regression analysis, we examined the assumptions of no multicollinearity and homoscedasticity. The variance inflation factor (VIF) for the independent variables ranged between 1.07 and 1.51 which was well below the rule of thumb that regards a VIF value <10, indicative of inconsequential collinearity [18]. There was no evidence of heteroscedasticity upon visual inspection of the plot of residuals (the difference between the observed and predicted values of dependent variables) against predicted values.

### Ethics

The study was performed as part of a larger study to develop management principles for chronic pain in the TSCI population. The study gained ethics approval from the University of the Western Cape’s Biomedical Research and Ethics Council (ethics approval: BM20/8/22). This research project was registered on the National Research Database (WC_202104_041). Permission to access the medical records was obtained from the Research Ethics Committee and Chief Executive Officer (CEO) of the rehabilitation centre. Participants’ details were saved on a password-protected document for the researcher and research assistants to access. Data was de-identified for statistical analysis to take place.

## Results

A total of 3601 medical records of persons admitted to WCRC for the above-mentioned period were drawn by the medical records personnel. Six hundred and forty records met the inclusion criteria; however, during the screening process, 15 files were not accessible due to various reasons such as missing files, archived files sent to a different location, files sent to autopsy, and one file was not accessible as the person was admitted to the in-patient facility. This resulted in 625 records of participants diagnosed with TSCI who had been admitted to the WCRC. Of those, 50 (8%) were deceased, either during their rehabilitation stay or once discharged.

### Gender, etiology and ISNCSCI

The characteristics indicated that males and participants aged 31–40 years were most prominent. These variables are noted in Table 1. Participants had a mean (SD) age of 38.0 years (*12.1*), with a mean (SD) LORS of 99 days (*130.9*). The most prevalent cause of injury in the cohort pertained to assault (predominantly assault via gunshot or stabbing) (70.4%). The thoracic spine was the most affected region (52.4%), with complete injuries (AIS A) being the most prevalent neurological classification (42.8%), according to the ISNCSCI. A notable limitation was the absence of AIS documentation for 29% of the cohort.

**Table 1:** ISCoS core data set.

Variable	Categories	N	%
	15–29	155	24.8
	30–44	325	51.9
	45–59	104	16.6
	60–74	27	4.3
	75+	10	1.6
Gender	Men	561	89.6
	Women	64	10.2
Date of injury (year)	2016	123	19.6
	2017	146	23.3
	2018	133	21.2
	2019	129	20.6
	2020	88	14.1
Severity and level of SCI	C1-4 AIS A, B and C	55	8.8
	C5-8 AIS A, B and C	52	8.3
	T1-S3 A, B and C	278	44.5
	AIS D at any injury level	55	8.8
Spinal region affected	Cervical	223	35.6
	Thoracic	328	52.4
	Lumbar	72	11.5
	Not documented	1	0.2
Spinal cord injury etiology	Assault	441	70.4
	Sports	4	0.6
	Fall	46	7.3
	Transport	119	19.0
	Other	8	1.3
	Unknown	1	0.2
AIS (ISNCSCI)	A	268	42.8
	B	72	11.5
	C	43	6.9
	D	55	8.9
	E	5	0.7
	Not documented	182	29.1
Vertebral injury	Yes	449	71.7
	No	174	27.8
	Not documented	3	0.5
Spinal surgery	Yes	115	18.4
	No	509	81.3
	Unknown	2	0.3

Table 1 consists of demographic and injury characteristics, according to the ISCoS core data set.

*AIS* (American Spinal Injury Association Impairment Scale), AIS A (motor and sensory complete); AIS B (motor complete and sensory incomplete); AIS C (motor and sensory incomplete with more than half of key muscles possessing a strength <grade 3; AIS D (motor and sensory incomplete with more than half of key muscles possessing a strength equal/> grade 3). Spinal cord injury etiology: Other refers to “*jump from height (not suicide); hit by forklift; trolley fell on person”. LORS: Length of Rehabilitation stay.*

### Length of rehabilitation stay

Table 2 contains the length of rehabilitation stay results, grouped by AIS classification, spinal region affected, the presence of a vertebral injury and surgery as well as spinal cord etiology, following the one-way ANOVA and t-test analyses.

**Table 2:** Length of rehabilitation stay (*one-way ANOVA and t-test*).

Variable	Mean stay	SD	F/t value	P	Effect size
AIS Classification			1.22	0.30	0.01
A (*N* = 268)	105.89	124.59			
B (*N* = 72)	124.66	167.47			
C (*N* = 43)	69.12	21.29			
D (*N* = 55)	116.52	219.05			
E (*N* = 5)	60.80	13.61			
Spinal region affected			9.78	<0.001	0.03
Cervical (*N* = 223)	128.90	172.51			
Thoracic (*N* = 328)	86.52	106.35			
Lumbar (*N* = 72)	65.76	33.65			
Vertebral injury			1.62	0.11	0.15
No (*N* = 174)	85.51	99.27			
Yes (*N* = 449)	104.51	141.26			
Surgery			0.47	0.64	0.05
No (*N* = 509)	98.00	132.35			
Yes (*N* = 115)	104.42	125.40			
Spinal cord etiology			0.98	0.38	0.003
Assault (*N* = 441)	101.8	143.30			
Fall (*N* = 46)	73.24	37.95			
Transport (*N* = 119)	97.49	100.89			

Table 2 contains the length of rehabilitation stay results, grouped by AIS classification, spinal region affected, the presence of a vertebral injury and surgery as well as spinal cord etiology, following the one-way ANOVA and t-test analyses.

The mean (SD) LORS for TSCI survivors in the rehabilitation centre was 99.0 days (*130.9*). The shortest stay was one day, and the longest was 1000 days. Most of the cohort stayed in the centre for 61—90 days (*N* = 46.2%). One-way ANOVA showed that the mean LORS was the highest for AIS B (Mean = 124.66, SD = 167.47) and lowest for AIS E (Mean = 60.80, SD = 13.61). The F-test demonstrated that there were no significant differences between the AIS classifications (F = 1.22, *p* = 0.300). The large standard deviations for all the categories show that there was a notable variability in the LORS.

One-way ANOVA demonstrated significant overall differences in LORS between the various spinal regions affected (F = 9.78, *p* < 0.001, η^2^ = 0.03). Levene’s F-test pointed out that the assumption of homogeneity of variance had been violated for the spinal regions affected groups (F = 15.33, *p* < 0.001) and thus the Games-Howell post-hoc test for unequal variances was used to compare the groups. According to the post-hoc analyses, those with cervical injuries stayed longer in the hospital than those with thoracic (mean difference = 42.16 *p* = 0.004) or lumbar injuries (mean difference = 63.12, *p* < 0.001). Those with thoracic injuries similarly stayed longer in hospital than those with lumbar injuries (mean difference = 20.96, *p* = 0.004).

With reference to age, Pearson r indicated that age was not related to LORS (r = 0.02, *p* = 0.67).

In terms of the presence of vertebral injuries and surgery, the two-sample t-tests revealed that there were no significant differences in LORS between those who had a vertebral injury and those who did not (t = 1.62, *p* = 0.11), and also between those who had spinal surgery and those who did not (t = 0.47, *p* = 0.64).

The linear regression confirms the results of the univariate analysis, and these can be found in Table 3. Factors such as age (young), male, without surgery and without a vertebral injury were associated with shorter LORS, however, not statistically significant.

**Table 3:** Multiple linear regression with length of rehabilitation stay as a dependent variable.

Predictor	Beta	SE	95% CI	Β	p-value
Gender	─12.97	23.106	[─55.08–35.76]	─0.02	0.58
Age	─0.56	0.61	[─1.69–0.71]	─0.05	0.35
Surgery	─25.91	18.80	[─62.86–11.04]	─0.073	0.17
Vertebral injury	25.99	17.22	[─7.86–59.85]	0.08	0.13
Spinal region affected – Thoracic*	─56.64	16.23	[─88.56–24.73]	─0.20	< 0.001
Spinal region affected – Lumbar*	─80.64	25.33	[─130.44–30.84]	─0.17	0.00
Etiology – transport^‡^	─6.14	17.97	[─41.46–29.17]	─0.018	0.73
Etiology – fall^‡^	─32.41	28.23	[─87.92–23.09]	─0.06	0.25
AIS B^¥^	15.74	19.52	[─22.63–54.12]	0.04	0.42
AIS C^¥^	─24.89	25.48	[─74.98–25.21]	─0.05	0.33
AIS D^¥^	12.39	21.95	[─30.76–55.53]	0.03	0.57

Table 3 presents the results of the multiple linear regression analysis examining gender, age, surgery, vertebral injury, spinal region affected, etiology, and AIS classification as predictor variables, with length of rehabilitation stay as the dependent variable.

*reference variable is cervical.

‡reference is assault.

^¥^reference is AIS A.

The multiple regression confirmed that a significant relationship between spinal region affected, and LORS (β = ─0.21, *p* < 0.001) existed. Persons with cervical injuries stayed longer in rehabilitation (mean = 128.90 days, SD = 172.51) than those who had thoracic (mean = 86.74 days, SD = 106.44) or lumbar (mean = 65.78 days, SD = 33.42, β = −0.18, *p* = 0.01) injuries. There were no significant differences in LORS and AIS, according to the ISNCSCI. There were trends to differences, but these did not meet statistical significance, such as persons with AIS C stayed fewer days than those with AIS A, and persons with AIS B and D stayed longer in the rehabilitation centre compared to those with AIS A.

## Discussion

The study aimed to examine the profile of persons with TSCI admitted to a rehabilitation centre and to describe the factors associated with the LORS, in the Cape Metropolitan region of the Western Cape, South Africa.

A few studies exist in South Africa examining the LORS. Alves, Pilusa [19] reported on the profile of persons with TSCI together with the mean LORS, in Gauteng, South Africa; however, their study did not include factors that affect or are associated with LORS. Joseph [20] found that participants’ age and LORS had an associated negative effect on the functional outcome of persons with TSCI. Our study filled the knowledge gap in providing factors associated with LORS, at a specialised rehabilitation centre in South Africa, and added to the existing body of knowledge by reporting on the current profile of individuals with TSCI.

Young males constituted the majority of the population in this study. Assault was the most prevalent cause of injury in the cohort. These findings parallel a previous study [5] which also found that young males were predominantly inflicted with TSCI due to assault in the Cape Metropolitan region. In the five years following the study conducted by Joseph and colleagues, little has changed in the Cape Metropolitan region. Studies conducted across LMICs and sub-Saharan countries (Ethiopia, Ghana, Nigeria, Senegal, Sierra Leone, South Africa and Zimbabwe) found that motor vehicle accidents were the most common etiology of TSCI, followed by falls and violence [2, 21]. Violence was particularly noted in South Africa. This finding is consistent with our results, where assault (predominantly by gunshot and stabbing) was the most prevalent etiology in Cape Town, South Africa.

Crime, including contact crimes, decreased in 2021/2022 [15], as reflected by a report on crime statistics in the Western Cape province of South Africa, which was published in 2022.The report further accredits the decrease in crime to the COVID-19 pandemic, in which lockdown levels imposed movement restrictions. There are ten police precincts attributing to most of the crime in the province, and six of those are located in the Cape Metropolitan region. Assault with intent to commit grievous bodily harm (GBH) was reported in 19452 cases, which displayed a decrease in the percentage of assault GBH cases in the Western Cape and nationally [15]. Despite the decrease in these numbers, our study still found assault amongst young males to be the highest cause of TSCI, which calls for an increase in crime prevention methods, particularly in the Cape Metropolitan region. The Western Cape government currently provides a range of programmes for youth at risk [22], such as the CASE and Western Cape Baptist organizations, to name a few, which provides a prevention programme for youth exposed to gang-violence. However, an increase in the promotion of these programmes may be required to address the increased cases of assault resulting in TSCI. These programmes should aim to further educate young males on the potential consequences of gunshot and stab injuries, emphasizing that survival of such acts of interpersonal violence may result in life-threatening conditions, including spinal cord injuries and traumatic brain injuries.

In our study, AIS A was the most prevalent injury severity, consistent with a similar study in the Gauteng province of South Africa [19]. A previous study performed in an acute hospital setting in South Africa also found the AIS A classification to be the most prevalent (6). Salient to rehabilitation planning, our study also found undocumented AIS in folders. The purpose of the ISNCSCI is to allow healthcare workers to set attainable and realistic goals in terms of physical rehabilitation for persons suffering from TSCI. The absence thereof reduces effective care [23].

The factors found in our study, which significantly predict LORS, indicated that persons with cervical injuries stayed longer in rehabilitation compared to those with thoracic or lumbar injuries, consistent with a study by Milicevic, Bukumiric [12] and Post, Dallmeijer [10]. These findings inform healthcare planning by highlighting the need for adequate resource allocation for individuals with cervical injuries, particularly ensuring access to the full interprofessional team, as such cases demand a high level of care due to injury severity and its impact on functioning. Studies conducted by Post, Dallmeijer [10], Milicevic, Bukumiric [12], Loganathan [24] correspond with our study in which we found that variables such as gender, age, vertebral injury, presence of spinal injury and AIS were not significant predictors of LORS. The LORS in our study was the longest for those with AIS B and the shortest for AIS E, which are similar to previous literature assessing predictors of LORS in persons with SCI/TSCI [10, 12, 24]. All these authors found that persons with complete injuries stayed longer in rehabilitation than those with incomplete injuries. These findings support the importance of documenting the ISNCSCI on admission to rehabilitation in order to effectively plan for healthcare, including factors such as bed availability and the allocation of staff.

The mean LORS found in our study is longer than the LOS for rehabilitations in Gauteng, South Africa, where the mean LORS equated to 85.59 [19]. One of the reasons for the increase in LORS in Cape Town may be that the Western Cape only has one government-funded specialised physical rehabilitation centre, contrary to the study conducted in Gauteng, which was conducted across five public rehabilitation centres. Our study found a huge variability in the LORS, and possible reasons could be the lack of planning by healthcare professionals due to missing AIS classifications documented and the lack of personalised rehabilitation resulting in a range of LORS.

Our study found that the LORS is shorter than that of European countries and one documented Asian country. This may be due to the higher percentage of reported prevalence of TSCI in South Africa, and particularly, in Cape Town, compared to European countries. Joseph, Delcarme [5] reported a crude incidence of 75.6 per million for TSCI in Cape Town, whereas Mitchell, Nunnerley [25] reported an incidence of 22 per million in the Netherlands. The demand for rehabilitation may be higher in Cape Town, and therefore, the length of rehabilitation may be strategically aligned to cater for this demand. In the Netherlands [10, 26], two separate studies documented persons with TSCI for a rehabilitation stay of 273 and 154 days. Possible influences on the higher reported LORS in the Netherlands are the cohort of patients included in the study (patients with poorer functional outcomes), the fact that readmissions to the acute hospital were ignored, and geriatric patients (who are discharged to the nursing home earlier) were excluded from the study [10]. The health insurance in the Netherlands provides no restrictions on the LORS [10]. In Italy, 143–164 (SD = 89.1) days were reported [27, 28], 217 days in Norway [28] and 239 (SD = 168) days for persons with TSCI in Israel [29]. The LORS in Israel was similar to earlier studies conducted in Europe, which may indicate that the LORS is usually longer in European countries as contrasted with American countries [29]. Longer rehabilitation stays of five to eight months results in improved outcomes for persons with poorer function at admission [29]. In the United States, a mean LORS of 60 (SD = 387) days was reported [30].

There is a clear need for patient-centered care considering the unique profile of TSCI in Cape Town, South Africa. Young males with assault as the predominant etiology requires the specific allocation of healthcare professionals, such as social workers and psychologists to aid in the comprehensive rehabilitation of these individuals. However, staff resources, particularly psychologists are scarce in the government-funded rehabilitation, in Cape Town, South Africa [31].

The study has a few limitations. The study only included adults and therefore further studies should include paediatric cases. The medical records were screened only at a rehabilitation centre in the public sector, and therefore future studies should include cases in the private rehabilitation sector and compare the two settings. Data regarding secondary medical complications, mental health clinical diagnoses, along with functional independence (motor-sensory scores) were not collected, which may also influence LORS.

## Conclusion

Young males in the 30-–44-year-old category were the most prevalent in the rehabilitation population, with assault as the dominant etiology. The factor associated with LORS was the spinal region affected. Consequently, persons with cervical injuries stayed significantly longer in rehabilitation compared to those with thoracic and lumbar spinal sections affected. In our study, the non-significant but worthwhile finding indicates that the LORS was the longest for those with AIS B and the shortest for AIS E, highlighting the importance of documenting the ISNCSCI at admission to rehabilitation, to aid healthcare planning, such as bed availability and the allocation of staff. The overall findings indicate a need to strengthen and promote existing prevention strategies in the Western Cape to address the high incidence of assault-related TSCI among young males.

Future studies should incorporate paediatric cases including private rehabilitation centres in Cape Town, South Africa. Studies should also investigate the reasons for variability in LORS by including a broader explanatory model of factors that could potentially influence LORS, such as secondary medical complications development, functional trajectories and number of goals set, to aid healthcare planning and optimise care.

## Data Availability

The datasets generated and analysed during the current study are not publicly available due to patient confidentiality but are available from the corresponding author on reasonable request.
